# Systems of nonlinear algebraic equations with positive solutions

**DOI:** 10.1186/s13660-017-1454-4

**Published:** 2017-08-01

**Authors:** Anca Ciurte, Sergiu Nedevschi, Ioan Rasa

**Affiliations:** 1 Department of Computer Science, Technical University of Cluj Napoca, 26-28th G. Baritiu Street, Cluj-Napoca, 400027 Romania; 2 Department of Mathematics, Technical University of Cluj Napoca, 26-28th G. Baritiu Street, Cluj-Napoca, 400027 Romania

**Keywords:** nonlinear algebraic system, positive solution, existence, uniqueness

## Abstract

We are concerned with the positive solutions of an algebraic system depending on a parameter $\alpha> 0$ and arising in economics. For $\alpha> 1$ we prove that the system has at least a solution. For $0<\alpha<1$ we give three proofs of the existence and a proof of the uniqueness of the solution. Brouwer’s theorem and inequalities involving convex functions are essential tools in our proofs.

## Introduction

Algebraic systems with positive solutions appear in a large variety of applications. Nonlinear systems of the form $F(x)=Ax+p$, $x \in\mathbb {R}^{n}_{+}$, where *A* is a positive matrix and *p* a non-negative vector, are investigated in [1]. Generalizing some results from [2], the existence of a positive solution is proved with Brouwer’s theorem, and the uniqueness is a consequence of suitable inequalities. Algorithms for finding the solution are also presented in [1]. Several problems can be converted into systems of this form (see also [2]): second order Dirichlet problems, Dirichlet problems for partial difference equations, third and fourth order difference equations, three point boundary value problems, steady states of complex dynamical networks, etc.

Extending the results of [1], systems of the more general form $\gamma_{i}(x_{i}) = \sum_{j=1}^{n} g_{ij}(x_{j})$, $1\leq i \leq n$, are studied in [3], together with a supplementary list of applications. The existence of a positive solution is proved by using a monotone iterative method; the proof of the uniqueness is based on an extension of the method used in [1].

Several classes of other systems and several methods to investigate the existence/uniqueness of their positive solutions are described, together with applications, in [3–13] and the references therein.

In this paper we consider an algebraic system which appears in some problems from economics, for example in establishing uniqueness of equilibrium of some models of trade with increasing returns: see [14].

Our proofs use Brouwer’s fixed point theorem and properties of minimum points of convex functions. Several inequalities, in particular inequalities related to convexity, are instrumental in these proofs.

The system addressed in this paper is described in what follows. Let $N\geq2$ be an integer. Given the real numbers $\alpha> 0$, $a_{ni}>0$, $b_{n}>0$, $i,n \in\{1,\dots, N\}$, consider the system of equations

1 $$ \sum_{n=1}^{N} b_{n} a_{ni} t_{i}^{\alpha- 1} \Biggl(\sum _{j=1}^{N} a_{nj} t_{j}^{\alpha}\Biggr)^{-1} = 1, \quad i=1,\dots,N. $$

We are interested in solutions $t=(t_{1}, \dots, t_{N}) \in D$ where $D:= \{t\in\mathbb{R}^{N} | t_{1}>0, \dots, t_{N}>0 \}$.

The existence and uniqueness of such a solution depend essentially on *α*. We shall see that for $\alpha\neq1$ there exists at least one solution; moreover, for $0<\alpha<1$ the solution is unique. For $\alpha=1$ there exist simple examples of systems (1) with no solution in *D*, or with exactly one solution, or with several solutions in *D*.

## The case $\alpha\neq1$: existence of the solution

### Theorem 1

*If* $\alpha> 0$, $\alpha\neq1$, *the system* (1) *has at least one solution in* *D*.

### Proof

For $j\in\{2,\dots,N\}$, let $t_{j} = s_{j} t_{1}$. Then (1) is equivalent to the system

2 $$ \left \{\textstyle\begin{array}{l@{\quad}l} \sum_{n=1}^{N} b_{n} a_{n1} ( a_{n1} + \sum_{j=2}^{N} a_{nj} s_{j}^{\alpha})^{-1} = t_{1}, &\\ \sum_{n=1}^{N} b_{n} a_{ni} s_{i}^{\alpha-1} ( a_{n1} + \sum_{j=2}^{N} a_{nj} s_{j}^{\alpha})^{-1} = t_{1}, & i=2,\dots,N, \end{array}\displaystyle \right . $$

with respect to the unknowns $t_{1}>0$, $s_{2}>0$, …, $s_{N}>0$.

From (2) we infer that $s_{2},\dots,s_{N}$ satisfy

3 $$ \begin{aligned}[b]& s_{i}^{\alpha-1} \sum_{n=1}^{N} b_{n} a_{ni} \Biggl( a_{n1} + \sum _{j=2}^{N} a_{nj} s_{j}^{\alpha}\Biggr)^{-1}\\&\quad = \sum_{n=1}^{N} b_{n} a_{n1} \Biggl( a_{n1} + \sum _{j=2}^{N} a_{nj} s_{j}^{\alpha}\Biggr)^{-1}, \quad i=2,\dots,N. \end{aligned}$$

Let $s:=(s_{2}, \dots, s_{N})$ and, for $i=2, \dots, N$,

$$\begin{aligned} F_{i}(s) :={}& \Biggl( \sum_{n=1}^{N} b_{n} a_{ni} \Biggl( a_{n1} + \sum _{j=2}^{N} a_{nj} s_{j}^{\alpha}\Biggr)^{-1} \frac{a_{n1}}{a_{ni}} \Biggr)^{1/(\alpha -1)} \\&\times \Biggl( \sum_{n=1}^{N} b_{n} a_{ni} \Biggl( a_{n1} + \sum _{j=2}^{N} a_{nj} s_{j}^{\alpha}\Biggr)^{-1} \Biggr)^{-1/{(\alpha-1)}}. \end{aligned}$$

Denote $F:=(F_{2}, \dots, F_{N})$; then (3) is equivalent to

4 $$ F(s) = s. $$

Let $U_{i} := \{ \frac{a_{n1}}{a_{ni}} | n=1,\dots,N \}$, $m_{i} := \min U_{i}$, $M_{i} = \max U_{i}$, $i=2,\dots,N$.

Then $F_{i}^{\alpha-1}(s)$ is a weighted mean of the numbers from $U_{i}$, and so

5 $$ m_{i} \leq F_{i}^{\alpha-1}(s) \leq M_{i}, \quad i=2,\dots,N, $$

for all $s=(s_{2},\dots, s_{N})$ such that $s_{2}>0$, …, $s_{N}>0$.

If $\alpha>1$, let $V:=\prod_{i=2}^{N} [m_{i}^{1/(\alpha-1)}, M_{i}^{1/(\alpha-1)}]$; if $0<\alpha<1$, let $V:=\prod_{i=2}^{N} [M_{i}^{1/(\alpha-1)}, m_{i}^{1/(\alpha-1)}]$.

In any case, due to (5) we can consider the continuous function $F:V\to V$. Since *V* is compact and convex, Brouwer’s theorem guarantees the existence of a solution $s^{0}\in V$ to (4). Then $s^{0}_{2}, \dots, s^{0}_{N}$ will satisfy (3). Now let

$$t_{1}^{0} := \sum_{n=1}^{N} b_{n} a_{n1} \Biggl( a_{n1} + \sum _{j=2}^{N} a_{nj} \bigl(s_{j}^{0} \bigr)^{\alpha} \Biggr)^{-1}. $$

Then $(t_{1}^{0}, s_{2}^{0}, \dots, s_{N}^{0})$ is a solution to (2), and consequently $t^{0} := (t_{1}^{0}, s_{2}^{0}t_{1}^{0}, \dots, s_{N}^{0}t_{1}^{0})\in D$ is a solution to (1). □

In the next example we present a system (1) with three solutions in *D*.

### Example 2.1

Let $N=2$, $\alpha=2$, $b_{1} = b_{2} = 1$, $a_{11} = a_{22} = 6$, $a_{12}=a_{21} = 1$. Then (1) becomes

6 $$ \left \{\textstyle\begin{array}{l} \frac{6t_{1}}{6t_{1}^{2}+t_{2}^{2}} + \frac{t_{1}}{t_{1}^{2}+6t_{2}^{2}} =1, \\ \frac{t_{2}}{6t_{1}^{2}+t_{2}^{2}} + \frac{6t_{2}}{t_{1}^{2}+6t_{2}^{2}} =1, \end{array}\displaystyle \right . $$

and has the solutions $(1,1)$, $(\frac{8}{7}, \frac{6}{7} ), (\frac{6}{7}, \frac{8}{7} ) \in D$.

## The case $\alpha=1$

In the next three examples we provide systems (1) with (i) one solution in *D*, (ii) no solution in *D*, (iii) infinitely many solutions in *D*. In each example we take $N=2$, $\alpha=1$.

### Example 3.1

Let $a_{11} = a_{21}=\frac{1}{2}$, $a_{12}=\frac {1}{3}$, $a_{22}=\frac{2}{3}$, $b_{1}=2$, $b_{2}=3$. The corresponding system (1) has a unique solution in *D*, namely $(2,3)$.

### Example 3.2

Let again $a_{11} = a_{21}=\frac{1}{2}$, $a_{12}=\frac{1}{3}$, $a_{22}=\frac{2}{3}$, but now $b_{1}=2$, $b_{2}=1$. Then the system has no solution in *D*.

### Example 3.3

Let $a_{11} = a_{21}=a_{12}= a_{22}=b_{1}=b_{2}=1$. Then $(t,2-t)$ is a solution of (1) for all $0< t<2$.

## The case $0<\alpha<1$: existence and uniqueness of the solution

We begin with another proof of the existence of solution in *D* to the system (1) if $0<\alpha<1$.

Fix an $\epsilon>0$ sufficiently small, such that $N\epsilon\leq b_{1}+\cdots+b_{N}$ and

$$\frac{\epsilon^{\alpha-1}}{ ( b_{1}+ \cdots+b_{N} -(N-1)\epsilon )^{\alpha}} \geq\max \Biggl\{ \Biggl( \sum_{n=1}^{N} b_{n}a_{ni} \Biggl( \sum_{j=1}^{N} a_{nj} \Biggr)^{-1} \Biggr)^{-1} \Big| i=1, \dots,N \Biggr\} . $$

Let $W:= \{ t\in\mathbb{R}^{N} \text{ } | \text{ } \sum_{j=1}^{N} t_{j} = \sum_{j=1}^{N} b_{j} , \text{ }t_{j}\geq\epsilon, \text{ } j = 1,\dots , N \}$. For $t\in W$ and $i\in\{ 1,\dots,N\}$ let $H_{i}(t) := \sum_{n=1}^{N} b_{n} a_{ni} t_{i}^{\alpha}( \sum_{j=1}^{N} a_{nj} t_{j}^{\alpha} )^{-1}$.

It is not difficult to verify that $H_{i}(t)\in W$, so that we can consider the continuous function $H=(H_{1}, \dots, H_{N}):W\to W$. Since *W* is compact and convex, from Brouwer’s theorem we deduce the existence of a $t\in W$ such that $H(t) = t$. Obviously $t\in D$ and *t* is a solution to (1).

The next theorem offers a third proof of the existence and also a proof of the uniqueness of the solution to (1) in the case $0<\alpha<1$.

### Theorem 2

*If* $0<\alpha<1$, *the system* (1) *has a unique solution in* *D*.

### Proof

Let $K:=\{ t\in\mathbb{R}^{N} | t\neq0, t_{1}\geq0, \dots, t_{N} \geq0\}$ and $f:K\to\mathbb{R}$, $f(t):= \alpha\sum_{n=1}^{N} t_{n} - \sum_{n=1}^{N} b_{n} \log\sum_{j=1}^{N} a_{nj} t_{j}^{\alpha}$.

The proof of the theorem is divided into the following steps.

*(1)* *f* *is strictly convex.*

Indeed, let $s,t \in K$, $s\neq t$. Since the function $u\to u^{\alpha}$ ($u\geq0$) is strictly concave, we have

$$\biggl( \frac{s_{j} +t_{j}}{2} \biggr)^{\alpha}\geq\frac{s_{j}^{\alpha}+t_{j}^{\alpha}}{2}, \quad j=1,\dots, N, $$

with at least one strict inequality.

Thus

$$\sum_{j=1}^{N}{a_{nj} \biggl( \frac{s_{j} +t_{j}}{2} \biggr)^{\alpha}} > \frac{1}{2} \Biggl( \sum _{j=1}^{N}{a_{nj}s_{j}^{\alpha}+ \sum_{j=1}^{N}{a_{nj}t_{j}^{\alpha}}} \Biggr), \quad n=1,\dots,N. $$

The function log is strictly increasing and strictly concave, so that

$$\begin{aligned} \sum_{n=1}^{N} {b_{n} \log\sum _{j=1}^{N}{a_{nj} \biggl( \frac{s_{j} +t_{j}}{2} \biggr)^{\alpha}}} &> \sum_{n=1}^{N}{b_{n} \log{\frac{1}{2} \Biggl( \sum_{j=1}^{N}{a_{nj}s_{j}^{\alpha}+ \sum_{j=1}^{N}{a_{nj}t_{j}^{\alpha}}} \Biggr)}} \\&>\frac{1}{2} \Biggl( \sum_{n=1}^{N} b_{n} \log{\sum_{j=1}^{N} {a_{nj}s_{j}^{\alpha}}} + \sum _{n=1}^{N} b_{n} \log{\sum _{j=1}^{N} {a_{nj}t_{j}^{\alpha}}} \Biggr). \end{aligned}$$

It follows immediately that

$$f \biggl(\frac{s+t}{2} \biggr) < \frac{1}{2} \bigl( f(s) + f(t) \bigr), $$

which means that *f* is strictly convex.

*(2)* $f:K\to\mathbb{R}$ *has a global minimum point* *x, and* $x\in D$ *.*

Let $\| t\| = (t_{1}^{2}+\cdots+t_{N}^{2})^{1/2}$. Obviously

7 $$ \lim_{\| t\|\to0} f(t)=+\infty. $$

Writing

$$f(t) = \sum_{j=1}^{N} \Biggl(\alpha t_{j} - \Biggl( \sum_{n=1}^{N} b_{n} a_{nj} \Biggr) t_{j}^{\alpha}\Biggr) + \sum_{n=1}^{N} b_{n} \Biggl( \sum_{j=1}^{N} a_{nj} t_{j}^{\alpha}- \log\sum_{j=1}^{N} a_{nj} t_{j}^{\alpha}\Biggr), $$

we see that

8 $$ \lim_{\| t\|\to+\infty} f(t)=+\infty. $$

For $0< r< R$, let $K_{r, R}:=\{t\in K \text{ }|\text{ } r\leq\|t\| \leq R \}$.

Let $s=(1,1,\dots,1)\in K$. Due to (7) and (8), there exist *r* and *R* such that, for all $t\in K \setminus K_{r, R}$,

9 $$ f(s) < f(t). $$

Since $K_{r,R}$ is compact, the continuous function *f* restricted to $K_{r,R}$ has a global minimum point in $K_{r,R}$; let us denote it by $x \in K_{r, R}$. Due to (9), $s\in K_{r,R}$, and so $f(x)\leq f(s) < f(t)$ for all $t \in K \setminus K_{r, R}$. It follows that *x* is a global minimum point of $f:K\to\mathbb{R}$.

Suppose that $x\notin D$. Then, for example, $x=(0,x_{2}, \dots, x_{N})$ and $x\neq0$. Consider the function $g(u) := f(u, x_{2}, \dots, x_{N})$, $u\geq0$. We get $g'(0) = -\infty$, and so *x* cannot be a minimum point of *f*. This concludes the proof of (2).

*(3) The critical points* $t\in D$ *of* *f* *are the solutions in* *D* *of the system* (1).

Indeed, for $t\in D$, $\frac{\partial f}{\partial t_{i}}(t) = 0$, $i=1,\dots, N$, is equivalent to (1).

*(4) The system* (1) *has a unique solution in* *D, namely the global minimum point* *x.*

As a minimum point of *f* in the open set *D*, *x* is a critical point, hence a solution of (1). Let $y\in D$ be another solution, $y\neq x$. Then *y* is a critical point of *f*, and *f* is convex; it follows that *y* is a global minimum point of *f* on *D*. (See, *e.g.*, [15], p. 14, Theorem 1.17.) Thus $f(y)=f(x)$; since *f* is *strictly* convex, we have

$$f \biggl( \frac{x+y}{2} \biggr) < \frac{f(x)+ f(y)}{2} = f(x). $$

This contradicts the global minimality property of *x* and the proof of Theorem 2 is finished. □

## References

[1] Ciurte A, Nedevschi S, Rasa I (2015). Systems of nonlinear algebraic equations with unique solution. Numer. Algorithms.

[2] Zhang G, Feng W (2007). On the number of positive solutions of a nonlinear algebraic system. Linear Algebra Appl..

[3] Győri I, Hartung F, Mohamady NA (2016). Existence and uniqueness of positive solutions of a system of nonlinear algebraic equations. Period. Math. Hung..

[4] Ciurte A, Nedevschi S, Rasa I (2012). An algorithm for solving some nonlinear systems with applications to extremum problems. Taiwan. J. Math..

[5] Bonanno G, Candito P, D’Agui G (2015). Positive solutions for a nonlinear parameter-depending algebraic system. Electron. J. Differ. Equ..

[6] Farina L, Rinaldi S (2000). Positive Linear Systems: Theory and Application.

[7] Kaykobad M (1985). Positive solutions of positive linear systems. Linear Algebra Appl..

[8] Nelson P (1972). Positive solutions of positive linear equations. Proc. Am. Math. Soc..

[9] Yang Y, Zhang JH (2008). Existence results for a nonlinear system with a parameter. J. Math. Anal. Appl..

[10] Yang Y, Zhang J (2009). Existence and multiple solutions for a nonlinear system with a parameter. Nonlinear Anal..

[11] Zhang G (2007). Existence of non-zero solutions for a nonlinear system with a parameter. Nonlinear Anal..

[12] Zhang G, Bai L (2009). Existence of solutions for a nonlinear algebraic system. Discrete Dyn. Nat. Soc..

[13] Zhang G, Cheng SS (2006). Existence of solutions for a nonlinear system with a parameter. J. Math. Anal. Appl..

[14] Costinota A, Rodriguez-Clareb A (2014). Trade theory with numbers: quantifying the consequences of globalization. Handbook of International Economics.

[15] Horst R, Pardalos PM, Thoai NV (1995). Introduction to Global Optimization.

